# The impact of treatment with bile acid sequestrants on quality of life in patients with bile acid diarrhoea

**DOI:** 10.1186/s12876-022-02404-9

**Published:** 2022-07-02

**Authors:** Aditi Kumar, Niall Galbraith, Hafid O. Al-Hassi, Manushri Jain, Oliver Phipps, Jeffrey Butterworth, Helen Steed, John McLaughlin, Matthew J. Brookes

**Affiliations:** 1 The Royal Wolverhampton NHS Trust, Wolverhampton Road, Wolverhampton, WV10 0QP UK; 2 Faculty of Science and Engineering, Research Institute in Healthcare Science, University of Wolverhampton, Wolverhampton, UK; 3 Shrewsbury and Telford Hospital NHS Trust, Shrewsbury, UK; 4 School of Medicine and Clinical Practice, Faculty of Sciences and Engineering, University of Wolverhampton, Wolverhampton, UK; 5 Division of Diabetes, Endocrinology and Gastroenterology, Faculty of Biology Medicine and Health, The University of Manchester, Manchester Academic Health Science Centre, Manchester, UK; 6 Department of Gastroenterology, Salford Royal Foundation Trust, Stott Lane, Salford, UK

**Keywords:** Bile acid diarrhoea, Crohn’s disease, Diarrhoea, Quality of life, Inflammatory bowel disease, Colesevelam

## Abstract

**Background:**

Bile acid diarrhoea (BAD) can be severely debilitating and negatively affect patients’ quality of life (QoL). We carried out a multi-centre prospective study exploring QoL outcomes in patients with BAD after treatment with colesevelam.

**Methods:**

Patients with or without a positive 23-seleno-25-homotaurocholic acid (SeHCAT) scan were recruited and categorised into four groups: SeHCAT negative control group (CG), idiopathic BAD, post-cholecystectomy (PC) and post-terminal ileal resection for Crohn’s disease (CD). Patients with a positive SeHCAT were treated with colesevelam and dosing was titrated to symptomatic response. Patients were reviewed at 4- and 8-weekly intervals and QoL was evaluated by EQ-5D-3L, SF-36, IBDQ-32 at each visit (where relevant). Patients with a negative SeHCAT (CG cohort) completed one set of questionnaires before being discharged from the study.

**Results:**

47 patients (BAD = 24, PC = 12, CD = 11) completed paired QoL questionnaires before and after treatment and 30 CG patients completed a baseline questionnaire. There was a significant improvement in IBDQ-32 mean scores before and after treatment in CD patients [134.6 (95%CI 112.5–156.6) and 158.4 (136.1–180.6), respectively (*p* = 0.007). Following treatment, BAD patients had significantly improved mean SF-36 scores in the “Role limitation due to physical health” dimension (*p* = 0.02) and in the overall mental component summary (*p* = 0.03). Prior to starting treatment, BAD patients had the lowest scores in the ‘activity’ dimension of the EQ-5D-3L (*p* = 0.04), which improved significantly after treatment (*p* = 0.002). Overall, the BAD and CD cohort showed improved mean scores with treatment in all components of the SF-36 and EQ-5D-3L, while the PC cohort showed a general decline in mean scores after treatment. 55% of patients clinically responded to treatment of which 41.7%, 58.3% and 81.8% responded from the BAD, PC and CD groups respectively. Correlations between those deemed as responders with improvements on the SF-36 and EQ-5D dimensions were not statistically significant.

**Conclusion:**

Our results demonstrate improved QoL in the BAD and CD cohort with treatment. Further larger studies are recommended specifically investigating the PC cohort and whether patients may improve with newer treatments such as FXR agonists.

*Trial registration* Ethical approval REC Ref: 16/LO/1325.

**Supplementary Information:**

The online version contains supplementary material available at 10.1186/s12876-022-02404-9.

## Background

Bile acid diarrhoea (BAD) is known to cause a wide spectrum of gastrointestinal symptoms, including intermittent or persistent diarrhoea, urgency, excessive flatulence, abdominal pain and faecal incontinence [1]. It is estimated that 1% of the population are affected by BAD [2], with approximately 10–30% of patients being misdiagnosed with diarrhoea predominant irritable bowel syndrome (IBS-D)[2, 3]. Unfortunately, there is poor recognition of this diagnosis by clinicians with diagnostic delay often exceeding five years and a large unmet need in symptom control despite treatment being available [4]. BAD can be classified into three types: Type 1 is secondary to ileal resection or ileal inflammation, with the most common cause being Crohn’s disease (CD); type 2 is idiopathic or primary BAD; and type 3 is secondary to various gastrointestinal diseases (cholecystectomy, small bowel intestinal bacterial overgrowth, coeliac disease, chronic pancreatitis) [5]. BAD has been shown to have a prevalence of greater than 90% of patients in post-cholecystectomy diarrhoea [6] and over 90% with a terminal ileal resection [7, 8].

In the UK, the gold standard diagnostic test is the ^75^selenium-homotaurocholic acid test (^75^SeHCAT) [9]. Selenium-75 homocholic acid taurine is a synthetic analogue of taurocholic acid and this substrate is ingested as a capsule after an overnight fast. An initial gamma measurement is taken after three hours and this value is then compared to the same measurement after 7 days. The second measurement is divided by the first to give the proportion of ^75^SeHCAT retained, expressed as a percentage [8]. The SeHCAT molecule undergoes the same enterohepatic circulation as natural BAs, thus tracking its retention and loss [5]. Unfortunately, the SeHCAT is not available globally and usually only accessible in tertiary centres, with most clinicians instead preferring a blind empirical trial of treatment to diagnose BAD [10]. Treatment is with bile acid sequestrants (BAS), namely cholestyramine, colesevelam and colestipol with each medication having its pros and cons. Although cholestyramine is first-line treatment, it is poorly tolerated due to the taste, texture and smell. Colesevelam and colestipol have greater documented compliance and fewer reported side effects but are more expensive [8].

Symptoms of BAD can be severely debilitating and negatively affect patients’ quality of life (QoL). A poor QoL has been shown to correlate with disability, healthcare resource utilisation and clinical response to treatment. A recent survey reporting on patient symptoms and outcomes with BAD documented that over 80% of patients had associated depression, isolation, helplessness and low self-esteem [4]. However, there are limited studies that have explored health-related QoL outcomes in people with BAD. Consequently, the National Institute for Health and Care Excellence has recommended that further research is needed in this area to determine what, if any, QoL improvements can be seen in patients with BAS therapy [9]. We have carried out a multi-centre prospective study exploring QoL outcomes in patients with BAD after treatment with BAS.

## Methods

### Study design

This was a multicentre study that ran across three sites within the United Kingdom, including the Royal Wolverhampton NHS Trust, the Royal Shrewsbury and Telford NHS Trust and the Royal Stoke University Hospital. Each site followed the same pre-approved study protocol and adhered to the principles of Good Clinical Practice.

### Patient recruitment

All patients over the age of 18 years and able to provide written consent who had a SeHCAT scan requested for ongoing symptoms of diarrhoea between January 1st 2017 to October 31st 2021 were screened for eligibility. Prior to having a SeHCAT scan requested, all patients underwent BSG guideline compliant screening for chronic diarrhoea with blood tests, coeliac screen, faecal calprotectin and where necessary, endoscopy and magnetic resonance imaging [11]. Patients were excluded if they were pregnant or breastfeeding, unable to provide written consent, already had an established diagnosis of BAD, or were given antibiotics 4 weeks prior to or during study recruitment. SeHCAT results were classified into mild, moderate and severe disease based on their retention values of < 15%, < 10% and < 5% respectively. If the retention score was between 15 and 20%, patients were classed as indeterminate but were still given a trial of treatment. Patients were then categorised into four groups: SeHCAT negative control group (CG); idiopathic BAD, post-cholecystectomy (PC) and terminal ileal Crohn’s disease (CD). Prior to being diagnosed with BAD, patients with CD were excluded from having active disease with either a colonoscopy and/or faecal calprotectin levels. Patients with a positive or indeterminate SeHCAT result received a therapeutic trial of BAS. To ensure greater treatment adherence, colesevelam 625 mg once or twice daily, depending on symptoms was offered as first line treatment. All patients were given standard advice to maintain a 4-h window from taking their BAS to any of their other medication.

Patients were reviewed in a research clinic before treatment initiation and 4- and 8- weeks after treatment commencement. At each clinic appointment, patients were required to complete a 7-day stool chart prior to their appointment where daily stool frequency and consistency (Bristol Stool Form Scale) were documented. Three QoL questionnaires (EQ5D-3L, SF-36 and IBDQ-32) were completed prior to attending each clinic appointment. Clinical response was defined as patients who had improved bowel frequency by > 50% from their initial assessment or less than 3 bowel movements per day. If patients had a partial response (< 50% improvement from their initial assessment or > 3 bowel movements/day), their BAS dose was increased at their clinic appointment and reviewed in 4 weeks’ time. Any side effects of the treatment were documented, as well as review of their medication history. If patients could not tolerate the BAS or decided they did not want to continue in the study, they were removed from the study although the information collected up to that point was still used for analyses. The study was complete after 8 weeks of treatment and patients were referred back to their original clinician. Whilst we endeavoured to obtain completed questionnaires at 4- and 8-weeks to compare changes in QoL over time, not enough questionnaires were returned for meaningful analysis at 4-weeks. Thus, our results will be a QoL analysis after 8 weeks of treatment.

### Questionnaires

#### Short-form 36 (SF-36)

The SF-36 is a widely used QoL questionnaire that has been extensively used in both clinical and observational studies. This self-administered survey aims to review 8 dimensions of QoL including physical functioning (PF), role limitations due to physical (RP) and emotional health (RE), bodily pain (BP), general health (GH) perceptions, vitality (VIT), social functioning (SF) and emotional well-being (EB) [12]. Each scale is scored from 0 to 100, with the lower the score, the greater the disability. These 8 dimensions can then be combined to compute two summary scores reflecting overall health components: physical component summary (PCS) and mental component summary (MCS) [13]. The SF-36 is the most extensively evaluated generic patient reported outcomes questionnaire and has been validated for numerous purposes, including comparing disease burden or health states and comparing multiple treatments within a specific disease area [14, 15].

#### EQ-5D-3L

EQ-5D-3L is a versatile, generic assessment of QoL that provides an overview of general health related QoL by assessing 5 individual components alongside a visual analogue scale (VAS). The five dimensions are: mobility (MOB), self-care (SC), usual activities (ACT), pain and discomfort (PD) and anxiety and depression (AD). Each dimension has three levels: no problems, some problems and extreme problems. The patient is required to tick the box next to the most appropriate statement in each of the five dimensions, with their decision resulting in a 1-digit number. The VAS scale is a single 100-point scale which records the patient’s self-rated health with 0 being the ‘worst imaginable health state’ and 100 being the ‘best imaginable health state’ [16].

#### IBDQ-32

The IBDQ-32 is a 32-item self-administered measure of QoL specifically designed to assess patients with inflammatory bowel disease (IBD) in 4 key domains: bowel symptoms (loose stools, abdominal pain), systemic symptoms (fatigue, altered sleep patterns), emotional function (anger, depression, irritability) and social function (work attendance, need to cancel social events). All items use the 7-point Likert-type scales for capturing symptoms-related experiences over the previous 2 weeks, with 1 indicating the highest symptoms frequency and/or severity and 7 indicating the lowest symptom frequency and/or severity. The total score is calculated as the sum of all 32 items (scores ranging from 32 to 224). The higher the total score, the better the QoL [17]. This survey has been rigorously developed and validated for IBD patients, and responsive to disease activity changes [17, 18]. Clinical remission in patients with luminal CD has been shown to correspond to an IBDQ-32 score of 170 or higher [19] and meaningful change has been estimated at 16 points for the overall score [17, 18].

### Statistical analysis

The statistical level of significance for all tests was defined as *p* ≤ 0.05. We compared paired continuous data using two-tailed Student’s paired t-tests. defined as *p* ≤ 0.05. We compared paired continuous data using two-tailed Student’s paired t-tests. Comparisons between groups (CG, BAD, PC and CD) employed one-way ANOVAs. We performed statistical analyses using SPSS version 21 (SPSS, Chicago, IL, USA). Cohen’s d effect size was calculated to assess the magnitude of clinical effects for changes in QoL scores within each patient cohort before and after treatment. This standardised measure of change was calculated by dividing the difference between pre- and post-treatment scores by the standard deviation of the pre-treatment scores. Effect sizes were defined as small if 0.2, moderate if 0.5 and large if 0.8. This effect size was then used to calculate if a minimal clinical difference (MCD) was exceeded, using the recognised definition of an effect size greater than 0.2 [20].

## Results

**Fig. 1:**
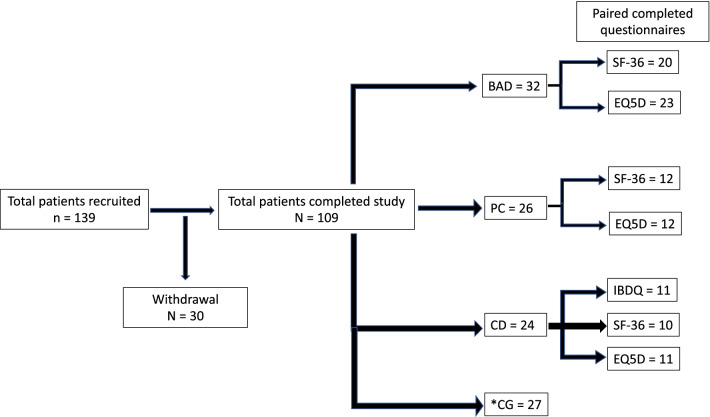
Patient recruitment into the four arms of the study. 139 patients were recruited into the study of which 109 patients completed a before and after visit. Following their SeHCAT results and based on their underlying pathology, they were categorised into either BAD (idiopathic bile acid diarrhoea), PC (post-cholecystectomy), CD (Terminal ileal resected Crohn’s Disease) and CG (SeHCAT negative control group). Of those patients that completed the study, 47 patients in total completed and returned their questionnaires for all visits. *CG group only had baseline questionnaires completed

139 patients were recruited into the study, of which 109 patients completed it. 71 patients completed the three distinct questionnaires in full (Fig. 1) giving a response rate of 65%. A total of 47 patients (BAD = 24, PC = 12, CD = 11) had a positive SeHCAT scan and completed before and after treatment questionnaires. The CG arm (n = 27) had normal SeHCAT scans and therefore only completed the initial questionnaires as per the rest of the three patient cohorts (pre-treatment). The CG cohort were not followed up after their negative SeHCAT scan. The mean age of participants was 50.4 years with 57.4% being female. A breakdown of patient demographics according to each group cohort can be viewed in the Additional file 1: Supplementary files.

### IBDQ-32

**Fig. 2:**
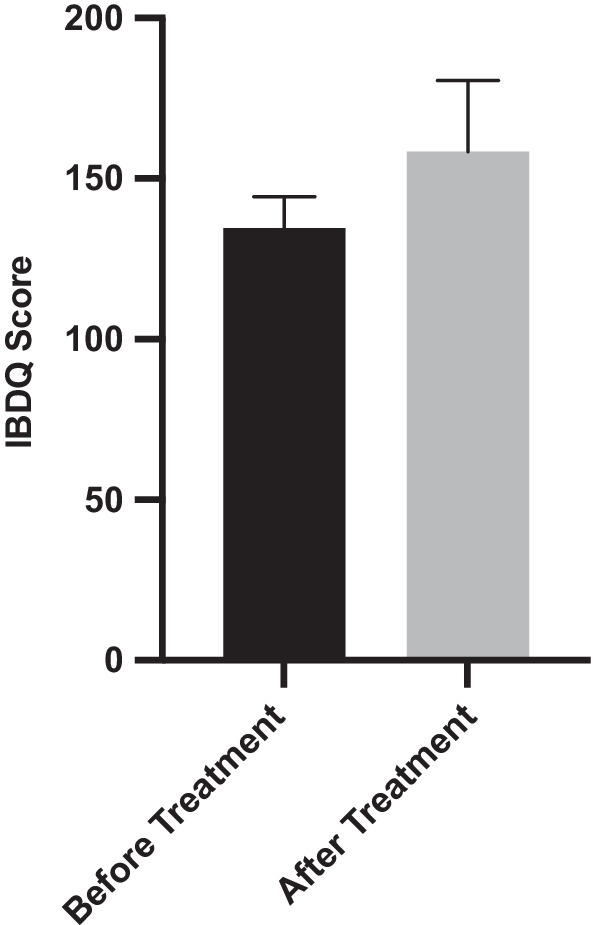
IBDQ-32 mean scores before and after treatment with colesevelam (*p* = 0.007). Error bars display standard error of the mean (SEM)

Of the 11 patients (CD only) who completed the IBDQ-32 in full, the mean age was 48.1 years with 63.6% females. There was a significant improvement in scores before and after treatment with mean scores of 134.6 (95%CI 112.5–156.6) and 158.4 (136.1–180.6), respectively (*p* = 0.007) (Fig. 2). All patients in the CD group had a SeHCAT retention score of < 5%, which is categorised as severe BAD. There was a moderate effect size before and after treatment of 0.7, which was clinically and statistically significant (*p* = 0.03) (Table 1).

**Table 1:** Comparison of patient groups who did and did not have an improvement in their quality of life pre and post treatment

	Grade of Effect size	Group	*p* Value
SF-36			
Physical component	Small	BAD	0.15
	Small	PC	0.37
	Small	CD	0.26
Mental component	Moderate	BAD	*0.03
	Null	PC	0.73
	Moderate	CD	0.07
EQ-5D-3L			
Mobility	Null	BAD	1.00
	Small	PC	0.34
	Small	CD	0.17
Self-care	Small	BAD	0.33
	Small	PC	0.34
	Small	CD	0.34
Activity	Moderate	BAD	*0.002
	Small	PC	0.44
	Null	CD	1.00
Pain & Discomfort	Small	BAD	0.16
	Null	PC	0.59
	Moderate	CD	0.10
Anxiety & Depression	Small	BAD	0.06
	Small	PC	0.19
	Small	CD	0.19
VAS	Null	BAD	0.48
	Small	PC	0.39
	Small	CD	0.26
IBDQ-32	Moderate	CD	*0.03

Effect size was calculated by dividing the difference between baseline and post-treatment scores by the standard deviation of baseline scores, where effect sizes of > 0.2 was considered small, > 0.5 moderate and > 0.8 large. Null effect sizes are those who did not achieve any improvement in their quality of life scores. * denotes *p* < 0.05

### SF-36

A total of 42 patients completed the SF-36 questionnaire, 20 patients from BAD, 12 from PC and 10 from the CD cohort. The median age was 53 with 57.1% female respondents. In the BAD and CD cohort, mean score improvements were seen with BAS treatment in all components (Table 2). In the PC cohort, apart from general health and emotional well-being, mean scores were reduced in all components after treatment. In all components of the SF-36, the CG cohort generally scored higher in their baseline scores than the other three groups.

**Table 2:** SF-36 mean scores for each group before and after treatment. BAD: bile acid diarrhoea, PC: post-cholecystectomy, CD: terminal ileal resected Crohn’s Disease

Field	Time	Control Group	BAD	PC	CD	*p* Value
Physical Functioning (PF)	Before	66.00 (52.78–79.23)	75.17 (63.18–87.15)	63.66 (42.97–84.34)	75.00 (55.26–94.74	0.60
	After		78.00 (66.63–89.37)	62.17 (42.87–81.47)	80.50 (65.19–95.81)	0.17
	*p* value		*p* = 0.27	*p* = 0.79	*p* = 0.32	
Role limitation due to physical health (RP)	Before	41.35 (23.75–59.94)	32.89 (13.61–52.18)	54.17 (26.38–81.96)	52.50 (19.36–85.64)	0.50
	After		53.95 (34.57–73.32)	35.42 (4.78–66.05)	52.50 (18.31–86.69)	0.50
	*p* value		*p = 0.02	*p* = 0.11	*p* = 1.00	
Bodily Pain (BP)	Before	52.60 (41.55–63.64)	47.5 (35.12–59.88)	46.46 (37.46–55.46)	57.50 (32.85–82.15)	0.71
	After		57.78 (45.53–70.02)	46.04 (26.25–65.83)	67.5 (50.89–84.11)	0.18
	*p* value		P = 0.13	P = 0.96	P = 0.33	
General Health (GH)	Before	44.60 (34.23–54.97)	43.47 (29.54–57.40)	45.42 (33.73–57.11)	40.00 (20.78–59.22)	0.96
	After		43.89 (33.85–53.93)	47.08 (31.03–63.14)	46.50 (28.26–64.74)	0.92
	*p* value		*p* = 0.92	*p* = 0.70	*p* = 0.11	
Vitality (VT)	Before	39.40 (29.60–49.20)	30.25 (18.04–42.46)	40.68 (27.80–53.56)	23.50 (11.22–35.78)	0.19
	After		38.5 (26.88–50.12)	37.27 (19.35–55.19)	30.50 (11.84–49.16)	0.72
	*p* value		*p* = 0.11	*p* = 0.61	*p* = 0.25	
Social Functioning (SF)	Before	69.79 (57.64–81.94)	56.75 (42.20–71.30)	67.71 (52.77–82.65)	58.75 (41.86–75.64)	0.41
	After		65.63 (52.63–78.62)	66.67 (48.33–85.01)	66.25 (47.38–85.12)	0.99
	*p* value		*p* = 0.11	*p* = 0.90	*p* = 0.11	
Role limitation due to emotional health (RE)	Before	58.67 (40.34–76.99)	45.00 (25.88–64.12)	52.78 (25.00–80.55)	53.33 (21.14–85.52)	0.78
	After		65.00 (45.74–84.26)	44.44 (14.04–74.85)	66.67 (36.93–96.41)	0.37
	*p* value		*p* = 0.08	*p* = 0.63	*p* = 0.17	
Emotional well-being (EB)	Before	64.96 (54.99–74.93)	56.13 (45.96–66.30)	59.33 (47.29–71.38)	60.80 (49.86–71.74)	0.56
	After		62.67 (52.39–72.14)	61.61 (43.58–79.64)	70.00 (56.90–83.10)	0.63
	*p* value		*p* = 0.27	*p* = 0.68	*p* = 0.15	
Physical component summary (PCS)	Before	51.20 (44.57–57.83)	50.21 (42.44–57.97)	52.42 (43.83–61.02)	56.79 (45.13–68.46)	0.94
	After		58.87 (51.82–65.91)	47.68 (37.50–57.86)	61.86 (51.03–72.69)	0.37
	*p* value		*p* = 0.15	*p* = 0.37	*p* = 0.26	
Mental component summary (MCS)	Before	58.09 (51.50–64.68)	47.03 (39.90–54.17)	55.43 (46.86–64.00)	49.10 (39.24–58.95)	0.46
	After		57.85 (50.90–64.80)	52.82 (42.48–63.16)	58.35 (47.94–68.77)	0.83
	*p* value		* p = 0.03	*p* = 0.73	*p* = 0.07	

95% CI is represented within parenthesis. The *p* values in the far-right column reflect group comparisons before treatment and after treatment respectively. * denotes *p* < 0.05

Collating the SF-36 scores into the two summary components, the physical and mental component summaries showed overall improvements in the BAD and CD groups, with a significant improvement in mental component summaries in the BAD group (*p* = 0.03). The effect size for post-treatment improvements in the physical component summary exceeded the mean clinical difference for the BAD group (t = − 1.54, *df* = 14, *p* = 0.15, d = − 0.40) and the CD group (t = − 1.19, *df* = 9, *p* = 0.26, d = − 0.37). The PC group showed a decline in physical health (t = 0.93, *df* = 11, *p* = 0.37, d = 0.27). Simple comparisons of scores before and after treatment showed a statistically significant improvement in the mental component summary which exceeded the mean clinical difference in the BAD group (t = − 2.29, *df* = 19, *p* = 0.03, d = − 0.51) (Table 1). Although not statistically significant, there was clinically significant improvement in the mental component summary in the CD group which also exceeded the mean clinical difference (t = − 2.02, *df* = 9, *p* = 0.07, d = − 0.64). There was a slight non-significant decline in mental functioning in the PC group (t =  < 1, *df* = 9, d = 0.11).

Sub-analysis of SF-36 mean scores before and after treatment with the degree of severity of BAD did not any significant change in patients diagnosed with mild, moderate, severe or indeterminate disease.

### EQ-5D-3L

A total of 46 patients completed the EQ5D questionnaire, 23 from BAD, 12 from PC and 11 from the CD cohort. The mean age was 51 years with 56.5% female respondents. Whilst all three cohorts showed an improvement in VAS scores after treatment, the PC cohort showed a decline in all other dimensions (Table 3). The BAD and CD cohort showed improved mean scores in all components post-treatment. In all components of the EQ5D questionnaire, the CG cohort generally had higher baseline scores than the other three groups.

**Table 3:** EQ-5D-3L mean score comparisons between groups

Field	Visit	Control group	BAD	PC	CD	*p* Value
Mobility (MOB)	Before Treatment	1.50 (1.22–1.78)	1.30 (1.10–1.51)	1.36 (1.02–1.70)	1.18 (0.91–1.45)	0.39
	After treatment		1.30 (1.06–1.55)	1.55 (1.19–1.89)	1.00 (1.00–1.00)	*0.04
	*p* value		*p* = 1.0	*p* = 0.34	*p* = 0.17	
Self-care (SC)	Before treatment	1.25 (0.99–1.51)	1.04 (0.95–1.13)	1.08 (0.90–1.27)	1.09 (0.89–1.29)	0.36
	After treatment		1.00 (1.00–1.00)	1.17 (0.91–1.41)	1.00 (1.00–1.00)	0.05
	*p* value		*p* = 0.39	*p* = 0.34	*p* = 0.34	
Activity (ACT)	Before treatment	1.67 (1.40–1.94)	2.04 (1.76–2.24)	1.50 (1.07–1.93)	1.45 (1.10–1.81)	*0.04
	After treatment		1.52 (1.20–1.80)	1.67 (1.35–1.98)	1.45 (1.10–1.81)	0.65
	*p* value		* p = 0.002	*p* = 0.44	*p* = 1.0	
Pain and Discomfort (PD)	Before treatment	1.96 (1.72–2.19)	1.96 (1.75–2.16)	1.92 (1.59–2.24)	2.00 (1.47–2.52)	0.99
	After treatment		1.78 (1.52–2.04)	2.00 (1.62–2.38)	1.55 (1.08–2.01)	0.23
	*p* value		*p* = 0.16	*p* = 0.59	*p* = 0.1	
Anxiety and Depression (AD)	Before treatment	1.79 (1.49–2.10)	1.87 (1.60–2.14)	1.67 (1.35–1.98)	1.91 (1.71–2.11)	0.75
	After treatment		1.61 (1.36–1.86)	1.92 (1.41–2.42)	1.64 (1.30–1.98)	0.37
	*p* value		*p* = 0.06	*p* = 0.19	*p* = 0.19	
Visual Analogue Scale (VAS)	Before treatment	66.68 (55.89–77.47)	59.23 (48.70–69.85)	54.67 (39.59–69.75)	64.09 (46.17–82.01)	0.53
	After treatment		63.14 (54.14–72.14)	59.33 (41.82–76.85)	71.64 (58.25–85.02)	0.41
	*p* value		*p* = 0.48	*p* = 0.39	*p* = 0.26	

BAD: Bile acid diarrhoea, PC: post-cholecystectomy, CD: terminal ileal resected Crohn’s Disease. 95%CI is represented within parenthesis. *p* Values on the right-hand column indicate comparison of mean scores between the groups at that specific moment in time. The *p* values in each row indicate comparison of mean scores between each group before and after treatment. * denotes *p* < 0.05

Simple within group comparisons (before treatment compared to after) showed no change in mobility for the BAD group (t = 0.00, *p* = 1.00, *df* = 22, d = 0.00) but a decline in the PC group which exceeded the MCD threshold (t = − 1.00, df = 10, *p* = 0.34, d = − 0.30). There was a clinically significant (but not statistically significant) improvement in mobility in the CD group (t = 1.49, *df* = 10, *p* = 0.17, d = 0.45).

On the self-care dimension, simple comparisons, although not statistically significant, showed clinically significant improvement in the BAD (t = 1.00, *df* = 22, *p* = 0.39, d = 0.21) and CD groups (t = 1.00, *df* = 10, *p* = 0.34, d = 0.30) but a clinically significant decline in the PC group (t = − 1.00, *df* = 11, *p* = 0.34, d = − 0.29).

With the Activity dimension, simple comparisons revealed a statistically and clinically significant post-treatment improvement in activity in the BAD group (t = 3.49, *df* = 21, *p* = 0.002, d = 0.74), a clinically significant decline in the PC group (t = − 0.80, *p* = 0.44, *df* = 8, d = − 0.23) and no change in the CD group (t = 0.00, *p* = 1.0, *df* = 11, d = 0.00).

For the pain and discomfort dimension, simple within-group comparisons showed that pain and discomfort improved post-treatment in the BAD group (t = 1.45, *df* = 22, *p* = 0.16, d = 0.30) and the CD group (t = 1.84, *df* = 10, *p* = 0.10, d = 0.55). However, there was no statistical or clinically significant change in the PC group (t = − 0.56, *p* = 0.59, *df* = 11, d = − 0.16).

The final EQ-5D-3L dimension was anxiety and depression, simple within-group comparisons showed clinically significative improvements in both the BAD group (t = 2.02, *df* = 22, *p* = 0.06, d = 0.42) and the CD group (t = 1.40, *df* = 10, *p* = 0.19, d = 0.42) but with a clinically significant worsening of anxiety/depression in the PC group (t = − 1.39, *df* = 11, *p* = 0.19, d = − 0.40).

On the VAS, simple comparisons showed no statistically or clinically significant improvement in imagined health state in the BAD group (t = − 0.72, *p* = 0.48, *df* = 21, d = − 0.15), but did demonstrate a clinically significant improvement in both the PC group (t = − 0.90, *p* = 0.39, *df* = 11, d = − 0.26) and the CD group (t = − 1.19, *df* = 10, *p* = 0.26, d = − 0.36).

Sub-analysis of EQ-5D-3L mean scores before and after treatment with the degree of severity of BAD did not show any significant change between in patients diagnosed with mild, moderate, severe or indeterminate disease.

### Response to treatment

In total, 26 out of 47 (55%) patients clinically responded to treatment and when broken down into each separate cohort, 41.7% (10/24), 58.3% (7/12) and 81.8% (9/11) responded from the BAD, PC and CD groups respectively. Breaking these results down into disease severity based on their SeHCAT scan, only 42.9% (3/7) with mild disease (SeHCAT < 15%) clinically responded to treatment, 66.7% (8/12) responded with moderate disease (SeHCAT < 10%) and 75% (15/20) responded with treatment to severe disease (SeHCAT < 5%). 8 patients had an indeterminate SeHCAT score (15–20%) where a trial of treatment was given, of which no patients had a clinical response. The IBDQ-32 scores could not be compared based on severity as all patients with CD had severe BAD (SeHCAT < 5%). Stool frequency improved from a mean of 5.7 (Standard deviation 3.3) bowel movements/day to 3.1/day (SD 1.8) (*p* < 0.0001) and stool consistency (as per Bristol stool chart) improved from a mean of type 5.4 (SD 1.4) to type 4.1 (SD 1.6) (*p* = 0.0069). The breakdown of stool frequency and consistency per patient cohort can be found in the supplementary files (Additional file 1: Table S2). Correlations between those deemed as responders with improvements on the SF-36 and EQ-5D dimensions were computed. None were statistically significant. The most notable relationships were with SF-36 MCS (r = 0.24, N = 40, *p* = 0.13), EQ-5D pain and discomfort (r = − 0.16, N = 46, *p* = 0.29) and EQ-5D-3L VAS (r = 0.16, N = 46, *p* = 0.29). No other coefficients were > − 0.08.

Out of 47 patients that were started on colesevelam, 10 had their dose escalated successfully while 14 patients had a failed attempt to increase their dose. A total of 13 patients experienced adverse events (AE), with 7 patients complaining of constipation, 3 with headaches, 2 with abdominal pain and 1 patient complaining of insomnia and muscle aches. Out of those patients that did not clinically respond, only 2 patients could not escalate their doses due to AE. 8 patients stopped their colesevelam (BAD = 6, PC = 2) with no response to treatment being the primary cause (n = 6) followed by constipation (n = 1). The remaining patient was taking multiple other medications and could not successfully increase their dose and maintain a four-hour window between their other medications and thus made the decision to stop their colesevelam.

## Discussion

Despite the small numbers in this study, there are some important findings to discuss. Overall, the BAD and CD cohort showed improvements in their self-administered QoL questionnaires, whilst the PC cohort showed worse outcomes with treatment. Specifically, in the mobility and self-care dimensions of the EQ-5D-3L, the PC group showed a statistically significant worse outcome post-treatment as compared to the BAD and CD cohort. This is an interesting finding because the PC group actually had a greater clinical response than the BAD group. Damsgaard et al. recently published their findings of long-term effects of BAS in patients with BAD over a 13-year period [21]. Their results showed that out of 377 patients, 64% documented they had a reduced QoL due to ongoing diarrhoea despite treatment and 50% reported their diarrhoea to be unaltered or worse than before diagnosis. However, their study did not differentiate findings between the types of BAD. Our results from the PC cohort may indicate that there are alternate mechanisms underlying their poor response compared to the other cohorts. Further research is needed to understand why the positive improvements in QoL are not seen specifically in the PC group and perhaps extended studies with alternative dosing regimes will allow us to understand and optimise treatment in these groups.

The cohort that seemed to have the greatest response both clinically and in relation to their QoL was the CD group. Our results are consistent with the literature, where Nyhlin et al. demonstrated an 86% response to cholestyramine in CD patients [22]. Despite our small numbers in the CD cohort, we demonstrated a significant improvement in the IBDQ-32 scores with treatment. Whilst their scores in the EQ-5D-3L and SF-36 improved after treatment, this did not reach statistical significance, which is more likely because of the small numbers in the cohort. Our results demonstrate the importance of investigating BAD in CD remission patients with ongoing diarrhoea as treating this simple condition can lead to significant improvements in patient’s symptoms and QoL.

There were significant changes seen in the BAD cohort in their MCS scores of the SF-36 questionnaire and the activity dimension of the EQ-5D-3L before and after treatment, which met the MCD. This was also seen in the CD cohort in their IBDQ-32 scores before and after treatment. Based on the definition of effect size, ‘small’ improvements were seen amongst all the cohorts in fifteen components across the broad aspects of QoL, and ‘moderate’ improvements in five of the components. The literature argues that a moderate effect size (> 0.5) is required to demonstrate a significant clinical change in QoL and this has been validated clinically [23]. Interestingly, only the BAD and CD cohorts showed ‘moderate’ effect size changes, which again raises the question as to why the PC cohort did not respond similarly.

Interestingly, the CG cohort demonstrated higher baseline mean scores than the other groups in 4 out of the 10 dimensions of the SF-36, specifically with the mental health elements of the questionnaire (mental component summary, emotional well-being, role limitation due to emotional health and social functioning). They had the lowest mean scores in the role limitation due to physical health, general health and vitality components. Whilst these patients were not formally diagnosed with IBS-D, their mean scores are consistent with similar studies exploring QoL in IBS patients [24–26]. Gralnek et al. compared SF-36 scores in patients with IBS-D and healthy controls and found that the former had significantly lower scores on each of the 8 SF-36 scales (*p* < 0.001) with the lowest mean scale scores in the vitality, role limitation due to physical health, bodily pain and general health components [25]. Comparing IBS-D patients with a concomitant diagnosis of BAD, Bousaba et al. have recently demonstrated that these patients are more likely to have a greater negative impact on bowel and somatic symptoms and QoL compared to IBS-D patients without BAD [27]. Studies have previously demonstrated that gastrointestinal symptom severity is significantly associated with physical, but not mental QoL whilst psychosocial and somatic symptom measures correlate more closely with mental health-related QoL [28]; and IBS patients are more likely to be associated with concomitant psychiatric, and somatic co-morbidities [29]. Without further information, it is difficult to conclude whether our CG cohort had greater extraintestinal symptoms, such as somatic or mental health symptoms (low mood, anxiety, chronic stress or fatigue), on top of the traditionally elicited gastrointestinal symptoms that resulted in worsening scores in the mental health QoL components. This, along with a follow-up QoL assessment would be beneficial in determining whether these patients have worse outcomes than patients with established BAD.

A systematic review demonstrated a dose–response relationship between severity of BAD and treatment response, with 96% of patients with severe, 80% of patients with moderate and 70% of patients with mild BAD responding to cholestyramine therapy [2]. Another older study demonstrated persistent symptoms in 81% of patients with BAD despite treatment with cholestyramine [30]. However, there were limitations with both studies, whereas the former study was not standardised and included objective and subjective reports of symptomatic improvements from patients, the latter had a very small recruitment number (n = 16). Comparatively, our results were much more modest with clinical response rates of 75%, 67% and 43% in severe, moderate and mild disease, respectively. Whilst it is possible that the degree of BAD does not necessarily correlate with patient symptoms, which was demonstrated in our study but also confirmed by Farrugia et al. [31], the difference in our results could also be due to the first-line medication we chose to prescribe in our study. Colestyramine and colestipol are often discontinued due to their adverse effects of constipation, bloating, nausea and abdominal cramps as well as their poor taste and texture of the resin powder [30]. Colesevelam is another BAS that is available in tablet form and is often better tolerated by patients. To avoid medication discontinuation and intolerance with colestyramine, we chose to treat our patients with colesevelam as first-line therapy in our study. However, studies have demonstrated that whilst colesevelam has been shown to create a firmer stool consistency, it does not necessarily improve stool frequency [32, 33] and rather colestyramine has been found to be superior in reducing the number of watery stools [34]. This may be a reason why we did not document a greater clinical response as we determined clinical improvement based on stool frequency rather than consistency. When we attempted to titrate medication to a higher dose, patients encountered side effects of constipation, bloating and headaches. Many patients were on multiple medications and this had to be factored in when attempting to increase dosages due to the risk of interacting and reducing efficacy of the other drugs. Thus, despite the benefits of BAS being cheap and easily available, their use has yet to be validated by quantitative data, with consensus on dosing and duration of treatment to be established [35]. The use of BAS can also lead to other non-negligible complications such as vitamin and fat malabsorption (vitamins A, D and K), risk of osteoporosis and coagulation abnormalities [35]. These patients may benefit from a trial of obeticholic acid, which acts by stimulating FGF19 thereby reducing bile acid synthesis, and has been shown to improve both stool frequency and stool consistency [36, 37].

Our study had its limitations. We had small numbers in each cohort and is likely why our results did not reach statistical significance. Although we had a good response rate for our questionnaires, there will still be an element of responder bias. Furthermore, QoL is highly subjective and can be influenced by many factors, including patients’ beliefs on QoL perception. QoL can also change over time and it would have been beneficial to have additional background information in our SeHCAT negative CG cohort and explore whether their QoL would have changed without treatment; this should be an area for further research to obtain a greater understanding of patients QoL perceptions. Lastly, our results may have been confounded by including our ‘indeterminate’ SeHCAT patients (15–20%) as BAD positive, of which 0/8 patients responded to treatment. Although generally BAD is diagnosed when SeHCAT retention is < 15%, results are on a continuous scale and the threshold used for a positive result can vary [38]. Whilst retention values above 20% are not considered indicative of BAD, there is a grey area where it is uncertain if patients will respond with a retention value of 15–20%. Thus, we decided to trial these patients with treatment to assess their response and it became clearly evident that these patients do not benefit with treatment.

Despite these limitations, this is the first study that compared QoL outcomes and clinical response rates in patients with BAD and also compared results between the types of BAD. Our results warrant further larger studies preferably with longer follow-up duration to specifically investigate the PC and CG cohort and whether patients may improve with different newer treatments such as FXR agonists.

## Supplementary Information

**Additional file 1: Supplementary Table 1** outlines the patient demographics of each group cohort. **Supplementary Table 2** provides details of the mean pre- and post-treatment stool frequency and consistency following treatment with bile acid sequestrants in the different patient cohorts with diagnosed bile acid diarrhoea.

## Data Availability

The clinical data for the present study will not be shared publicly as participants were informed at the time of providing consent that only researchers involved in the project would have access to the information provided. Please contact the corresponding author for further information.

## References

[1] Walters JRF (2020). Diagnosis and management of bile acid diarrhoea: a survey of UK expert opinion and practice. Frontline Gastroenterol.

[2] Wedlake L (2009). Systematic review: the prevalence of idiopathic bile acid malabsorption as diagnosed by SeHCAT scanning in patients with diarrhoea-predominant irritable bowel syndrome. Aliment Pharmacol Ther.

[3] Lovell RM, Ford AC (2012). Global prevalence of and risk factors for irritable bowel syndrome: a meta-analysis. Clin Gastroenterol Hepatol.

[4] Bannaga A (2017). How bad is bile acid diarrhoea: an online survey of patient-reported symptoms and outcomes. BMJ Open Gastroenterol.

[5] Walters JR, Pattni SS (2010). Managing bile acid diarrhoea. Therap Adv Gastroenterol.

[6] Sciarretta G (1992). Post-cholecystectomy diarrhea: evidence of bile acid malabsorption assessed by SeHCAT test. Am J Gastroenterol.

[7] Lenicek M (2011). Bile acid malabsorption in inflammatory bowel disease: assessment by serum markers. Inflamm Bowel Dis.

[8] Mottacki N, Simren M, Bajor A (2016). Review article: bile acid diarrhoea - pathogenesis, diagnosis and management. Aliment Pharmacol Ther.

[9] Guidance N. DG44: SeHCAT (tauroselcholic[75 selenium] acid0 for diagnosing bile acid diarrhoea. In NICE Guidance. 2021.

[10] Berti G (2021). Empirical trial or diagnostic tests for bile acid diarrhea? That is the question!. J Dig Dis.

[11] Arasaradnam RP (2018). Guidelines for the investigation of chronic diarrhoea in adults: British Society of Gastroenterology, 3rd edition. Gut.

[12] Brazier JE (1992). Validating the SF-36 health survey questionnaire: new outcome measure for primary care. BMJ.

[13] Ware JE, Sherbourne CD (1992). The MOS 36-item short-form health survey (SF-36). I. Conceptual framework and item selection. Med Care.

[14] Garratt A (2002). Quality of life measurement: bibliographic study of patient assessed health outcome measures. BMJ.

[15] C, MIN. Measuring health: A guide to rating scales and questionnaires. 2nd Edition ed. 1996. Oxford University Press, New York

[16] T.E. , Group (1990). EuroQol- a new facility for the measurement of health-related quality of life. Health Policy.

[17] Irvine EJ (1994). Quality of life: a valid and reliable measure of therapeutic efficacy in the treatment of inflammatory bowel disease. Canadian Crohn's Relapse Prevention Trial Study Group. Gastroenterology.

[18] Guyatt G (1989). A new measure of health status for clinical trials in inflammatory bowel disease. Gastroenterology.

[19] Gregor JC (1997). An evaluation of utility measurement in Crohn's disease. Inflamm Bowel Dis.

[20] Copay AG (2007). Understanding the minimum clinically important difference: a review of concepts and methods. Spine J.

[21] Damsgaard B (2018). Long-term effect of medical treatment of diarrhoea in 377 patients with SeHCAT scan diagnosed bile acid malabsorption from 2003 to 2016; a retrospective study. Aliment Pharmacol Ther.

[22] Nyhlin H, Merrick MV, Eastwood MA (1994). Bile acid malabsorption in Crohn's disease and indications for its assessment using SeHCAT. Gut.

[23] Norman GR, Sloan JA, Wyrwich KW (2003). Interpretation of changes in health-related quality of life: the remarkable universality of half a standard deviation. Med Care.

[24] Amouretti M (2006). Impact of irritable bowel syndrome (IBS) on health-related quality of life (HRQOL). Gastroenterol Clin Biol.

[25] Gralnek IM (2000). The impact of irritable bowel syndrome on health-related quality of life. Gastroenterology.

[26] Spiegel BM (2004). Clinical determinants of health-related quality of life in patients with irritable bowel syndrome. Arch Intern Med.

[27] BouSaba J, et al. Impact of bile acid diarrhea in patients with diarrhea-predominant irritable bowel syndrome on symptoms and Quality of Life. Clin Gastroenterol Hepatol. 2021. 10.1016/j.cgh.2021.11.035 PMC9166633 34871814

[28] Addante R (2019). Predictors of health-related quality of life in irritable bowel syndrome patients compared with healthy individuals. J Clin Gastroenterol.

[29] Singh P (2012). Psychiatric, somatic and other functional gastrointestinal disorders in patients with irritable bowel syndrome at a tertiary care center. J Neurogastroenterol Motil.

[30] Rössel P (1999). Prognosis of adult-onset idiopathic bile acid malabsorption. Scand J Gastroenterol.

[31] Farrugia A (2021). Rates of bile acid diarrhoea after cholecystectomy: a multicentre audit. World J Surg.

[32] Odunsi-Shiyanbade ST (2010). Effects of chenodeoxycholate and a bile acid sequestrant, colesevelam, on intestinal transit and bowel function. Clin Gastroenterol Hepatol.

[33] Beigel F (2014). Colesevelam for the treatment of bile acid malabsorption-associated diarrhea in patients with Crohn's disease: a randomized, double-blind, placebo-controlled study. J Crohns Colitis.

[34] Fernandez-Banares F (2015). Randomised clinical trial: colestyramine vs. hydroxypropyl cellulose in patients with functional chronic watery diarrhoea. Aliment Pharmacol Ther.

[35] Fani B (2018). Pros and Cons of the SeHCAT Test in Bile Acid Diarrhea: A More Appropriate Use of an Old Nuclear Medicine Technique. Gastroenterol Res Pract.

[36] Walters JR (2015). The response of patients with bile acid diarrhoea to the farnesoid X receptor agonist obeticholic acid. Aliment Pharmacol Ther.

[37] Oduyebo I, Camilleri M (2017). Bile acid disease: the emerging epidemic. Curr Opin Gastroenterol.

[38] Harmala S, ONP. Diagnostic Assessment Programme: SeHCAT (tauroselcholic [75 selenium] acid) for the investigation of bile acid diarrhoea. , N.I.f.H.a.C. Excellence, Editor. 2020.

