# Supplementary material for: The impact of treatment with bile acid sequestrants on quality of life in patients with bile acid diarrhoea
Source: BMC Gastroenterol. 2022 Jul 2;22:325. doi: 10.1186/s12876-022-02404-9 (PMC9250209; doi:10.1186/s12876-022-02404-9)
Supplement: Supplementary file 1 — Additional file 1: Supplementary Table 1 outlines the patient demographics of each group cohort. Supplementary Table 2 provides details of the mean pre- and post-treatment stool frequency and consistency following treatment with bile acid sequestrants in the different patient cohorts with diagnosed bile acid diarrhoea. [file 12876_2022_2404_MOESM1_ESM.docx]

|  | CG  (n=27) | BAD  (n=24) | PC  (n=12) | CD  (n=11) |
| --- | --- | --- | --- | --- |
| Age | 54 | 46 | 61 | 48 |
| Gender | | | | |
| Female | 74% (20) | 46% (11) | 67% (8) | 64% (7) |
| Male | 26% (7) | 54% (13) | 33% (3) | 36% (4) |
| Ethnicity | | | | |
| White British | 96% (26) | 87.5% (21) | 100% (12) | 90.9% (10) |
| Black Caribbean | 3.7% (1) | 0% (0) | 0% (0) | 0% (0) |
| Indian Asian | 0% (0) | 12.5% (3) | 0% (0) | 9.1% (1) |
| Co-morbidities | | | | |
| IHD | 7.4% (2) | 0% (0) | 8.3% (1) | 9.1% (1) |
| T2DM | 7.4% (2) | 4.2% (1) | 16.7% (2) | 0% (0) |
| Malignancy  (Remission) | 11.1% (3) | 8.3% (2) | 16.7% (2) | 9.1% (1) |
| Ulcerative Colitis | 0 | 12.5% (3) | 8.3% (1) | 0% (0) |
| Chronic pain | 22.2% (6) | 4.2% (1) | 8.3% (1) | 0% (0) |
| Depression/Anxiety | 22.2% (6) | 12.5% (3) | 0% (0) | 0% (0) |
| Others^a^ | 44.4% (12) | 33.3% (8) | 41.7% (5) | 18% (2) |
| Nil | 29.6% (8) | 45.8% (11) | 33.3% (4) | 72.3% (8) |
| Medications | | | | |
| Anti-depressants^b^ | 29.6% (8) | 25% (6) | 8.3% (1) | 0% (0) |
| Anti-spasmodics | 18.5% (5) | 8.3% (2) | 8.3% (1) | 0% (0) |
| PPI | 22.2% (6) | 37.5% (9) | 41.2% (5) | 0% (0) |
| Analgesics^c^ | 26% (7) | 12.5% (3) | 0% (0) | 0% (0) |
| Immunosuppressants | 0 | 4.2% (1) | 0% (0) | 27.3% (3) |
| Biologics | 0 | 4.2% (1) | 0% (0) | 18.2% (2) |
| Others^d^ | 44.4% (12) | 29.2% (7) | 41.7% (5) | 27.3% (3) |
| Nil | 18.5% (5) | 45.8% (11) | 25% (3) | 54.5% (6) |

*Supplementary Table 1: Patient demographics per group cohort.*

1. *Includes hypertension, asthma or chronic obstructive pulmonary disease not on medications, epilepsy, and hypothyroid disease.*
2. *Although generally prescribed for depression and/or anxiety, some patients were prescribed this for analgesic relief.*
3. *Includes co-codamol or other opioid derivative, non-steroidal anti-inflammatory agents, pregabalin or gabapentin.*
4. *Includes hypoglycaemics, anti-hypertensives, anti-epileptics, statins*

| **Treatment cohort** | **Mean bowel movements per day** | |  | **Mean consistency based on Bristol stool chart** | |  |
| --- | --- | --- | --- | --- | --- | --- |
|  | **Before Treatment** | **After treatment** | **p-value** | **Before treatment** | **After treatment** | **p-value** |
| CG | 4.4 (2.5) |  |  | 5.8 (0.9) |  |  |
| BAD | 5.6 (2.7) | 3.7 (1.7) | 0.0004 | 5.7 (1.2) | 4.2 (1.8) | 0.02 |
| PC | 6.0 (5.3) | 2.8 (2.2) | 0.07 | 4.6 (1.8) | 3.6 (0.9) | 0.30 |
| CD | 5.6 (2.0) | 2.0 (1.0) | <0.0001 | 5.5 (0.7) | 4.3 (1.7) | 0.39 |

Supplementary Table 2: Mean Stool frequency and consistency with standard deviation pre- and post-treatment with bile acid sequestrants in the different patient cohorts with diagnosed bile acid diarrhoea. *CG = SeHCAT negative control group, BAD = idiopathic bile acid diarrhoea, PC = post-cholecystectomy, CD = post-operative terminal ileal resected Crohn’s disease*
